# Is Work-Related Rumination Associated with Deficits in Executive Functioning?

**DOI:** 10.3389/fpsyg.2016.01524

**Published:** 2016-09-30

**Authors:** Mark Cropley, Fred R. H. Zijlstra, Dawn Querstret, Sarah Beck

**Affiliations:** ^1^School of Psychology, University of SurreyGuildford, UK; ^2^Faculty of Psychology and Neuroscience, Maastricht UniversityMaastricht, Netherlands

**Keywords:** work-related rumination, executive functioning, recovery, cognitive failures, psychological detachment

## Abstract

Work-related rumination, that is, perseverative thinking about work during leisure time, has been associated with a range of negative health and wellbeing issues. The present paper examined the association between work-related rumination and cognitive processes centerd around the theoretical construct of executive functioning. Executive functioning is an umbrella term for high level cognitive processes such as planning, working memory, inhibition, mental flexibility; and it underlies how people manage and regulate their goal directed behavior. Three studies are reported. Study I, reports the results of a cross-sectional study of 240 employees, and demonstrates significant correlations between work-related rumination and three proxy measures of executive functioning: cognitive failures (0.33), cognitive flexibility (-0.24), and situational awareness at work (-0.28). Study II (*n* = 939), expands on the findings from study 1 and demonstrates that workers reporting medium and high work-related rumination were 2.8 and 5 times, respectively, more likely to report cognitive failures relative to low ruminators. High ruminators also demonstrated greater difficulties with ‘lapses of attention’ (OR = 4.8), ‘lack of focus of attention’ (OR = 3.4), and ‘absent mindedness’ (OR = 4.3). The final study, examined the association between work-related rumination and executive functioning using interview data from 2460 full time workers. Workers were divided into tertiles low, medium, and high. The findings showed that high work-related rumination was associated with deficits in starting (OR = 2.3) and finishing projects (OR = 2.4), fidgeting (OR = 1.9), memory (OR = 2.2), pursuing tasks in order (OR = 1.8), and feeling compelled to do things (OR = 2.0). It was argued that work-related rumination may not be related to work demands *per se*, but appears to be an executive functioning/control issue. Such findings are important for the design and delivery of intervention programes aimed at helping people to switch off and unwind from work.

## Introduction

There are many definitions of the term rumination in the psychological literature but the general consensus is that rumination refers to the process of thinking deeply, meditating, pondering, or musing over something. The term rumination comes from the Latin ‘ruminare,’ meaning to ‘turn over in the mind,’ or to chew the cud. Within the occupational context, we define work-related rumination as the process of perseverative thinking or dwelling about problems and issues relating to work (Cropley and Zijlstra, 2011). For example, a worker may ruminate about completing an important project on time, or stress over a future meeting, or they may ruminate about something negative that was said to them by their line manager or colleague. Workers tend to ruminate over tasks that are unfinished (Syrek and Antoni, 2014; Syrek et al., 2016). No matter what the initial stressor was, individuals continue to think and perseverate over the issue during their free time (Brosschot et al., 2007).

Thinking about work issues when not at work is quite common. Research suggests that many individuals find it difficult to unwind and continue thinking about work post work. Indeed it has been estimated that up to 70% of workers ruminate or worry at one time or another about work issues (Gallie et al., 1998). It doesn’t really matter if people think about work post work, it really only becomes an issue if it starts to affect their health and wellbeing. On the one hand, thinking about work issues when not at work may be beneficial and could lead to new insights and solutions to problems at work; and or by simply reflecting about the positive aspects of the working day could lead to enhanced self-efficacy and mood (Meier et al., 2016). Thus, complete psychological detachment from work may not be necessary or even desirable, especially when thinking about work is under one’s own volition (Cropley and Zijlstra, 2011). On the other hand, when thinking about work is unwanted and outside of one’s control, this could compromise the recovery process and lead to ill health in the long-term (Querstret and Cropley, 2012; Sonnentag and Fritz, 2015).

In the occupational health literature it is now widely accepted that workers need to recover from the effects of work demands when not at work, in order to prevent long term health consequences (Zijlstra and Sonnentag, 2006). Recovery from work may be defined as the psycho-physiological unwinding after effort expenditure at work (Geurts and Sonnentag, 2006; Sonnentag and Fritz, 2007; Zijlstra et al., 2014). It is now thought that the recovery process appears to be largely influenced by the extent to which people manage to cognitively disengage (or disconnect) from their work demands and related thoughts when not at work (Sonnentag and Zijlstra, 2006; Sonnentag et al., 2008; Cropley and Zijlstra, 2011). Inadequate psychological recovery and work-related rumination, have been associated with a range of health problems, including cardiovascular disease (Suadicani et al., 1993), fatigue and sleep (Berset et al., 2011; Querstret and Cropley, 2012), and negative mood (Pravettoni et al., 2007). Studies, however, are needed to understand the cognitive mechanisms that influence the recovery process.

There are many theories as to why people ruminate, but less is known about the mechanisms involved. It has, however, been hypothesized that rumination may be associated with individual differences in executive functioning (Brinker et al., 2013). Executive functioning is a theoretical construct relating to a set of higher order cognitive processes that relate to how people manage and regulate their goal directed behavior. Executive functioning is an umbrella term for a wide range of cognitive processes and abilities including: planning, concentration, flexible thinking, problem-solving, self-awareness, and working memory (Miyake et al., 2000). Executive functioning is thought to involve communication across multiple brain regions and pathways and is primarily regulated by the prefrontal region of the brain, and rumination is reasoned to disinhibit these circuits (Brosschot et al., 2007; Esposito et al., 2014) by taking the prefrontal cortex temporarily ‘oﬄine’ (Ottaviani et al., 2009). Executive control defines the mechanism or system that coordinates these various regions and processes to complete a particular task or problem.

Previous research suggests that rumination reduces cognitive flexibility, by placing additional demands on a system which has limited resources (Davis and Nolen-Hoeksema, 2000; Watkins and Brown, 2002). People who ruminate may find it difficult to maintain their concentration, their focus, and mental control. Indeed the association between ruminative thinking and cognitive impairments has been demonstrated in a number of clinical and experimental studies (Davis and Nolen-Hoeksema, 2000; Joormann and Vanderlind, 2014). Research has shown that people who ruminate show deficits in their cognitive inhibitory system by finding it difficult to maintain their attention on task-relevant information (Carson et al., 2003; Joormann, 2004; Mor and Daches, 2015). That is, people who ruminate tend to find it difficult to stay focused on the task at hand. The deficits in performance of tasks that require executive control is not necessary or primarily due to the effects of mood (Watkins and Brown, 2002; Whitmer and Banich, 2007).

Much of this work has been conducted within the laboratory and to our knowledge no study has examined the role of rumination and executive control in the occupational context. Planning, maintaining focus, problem solving are crucial skills in the modern workplace, and it is important to examine the association between work-related rumination and executive functioning. It may be particularly difficult for workers who ruminate to perform operations that require sustained attention, concentration, and control given that rumination consumes resources that could otherwise be directed to the task at hand. Due to deficits in the attention, performance, and vigilance, individuals who ruminate may be at an increased risk of missing deadlines, making mistakes or even being involved in accidents at work. It is therefore important to understand the association between rumination and executive control in the work context. The present paper reports three independent studies that examined the association between work-related rumination and activities related to central executive functioning.

## Study 1: Work-Related Rumination and Executive Control

This initial exploratory study examined the association between work-related rumination and three proxy measures of executive control: cognitive failures, work situational awareness, and cognitive flexibility. Based on the theoretical rationale above, three tentative hypotheses were proposed.

H1: Work-related rumination will be positively correlated with cognitive failures.
H2: Work-related rumination will be negatively correlated with cognitive flexibility.
H3: Work-related rumination will be negatively correlated with work situational awareness.

### Method

Using the snowballing sampling procedure, 240 workers in full-time employment completed an online survey. To minimize the influence of job characteristics affecting the results, individuals were drawn from a variety of occupational settings. The total sample (*n* = 240; 70% Females) consisted of workers in full-time employment, and they completed a short online survey. The mean age for the sample was 34.3 years (range 17–64 years; *SD* = 13.95).

### Measures

#### Work Related Rumination

The affective rumination subscale of the work related rumination questionnaire was used to assess people’s level of work related thoughts outside of work (Cropley et al., 2012). Items are responded to on a 5-point Likert scale using the following response options; 1 = very seldom/never, 2 = seldom, 3 = sometimes, 4 = often, 5 = very often/always, e.g., “Are you troubled by work-related issues when not at work?.” A mean score is calculated with higher scores indicating greater rumination. This measure has been used in a number of previous studies (Querstret and Cropley, 2012; Querstret et al., 2016), and it has good reliability and validity (Cropley et al., 2012; Syrek et al., 2016). The Cronbach’s alpha reliability for this scale was 0.91.

#### Cognitive Failures

The cognitive failures questionnaire (CFQ; Broadbent et al., 1982) consists of 25 items relating to everyday cognitive failures that people may experience in their everyday life. Items are scored along a 5-point rating scale ranging from 1 = never, 2 = very rarely, 3 = occasionally, 4 = quite often, 5 = very often, e.g., “do you find yourself suddenly wondering whether you’ve used a word correctly?” The measure has good reliability and validity and is widely used (Broadbent et al., 1982; Merckelbach et al., 1999). The Cronbach’s alpha reliability for this scale was 0.91.

#### Cognitive Flexibility

Cognitive flexibility was assessed using the cognitive flexibility inventory (Dennis and Vander Wal, 2010). The scale consists of 20 items with questions such as “I like to look at difficult situations from many different angles,” and each item is rated on a 7-point scale; 1 = strongly disagree, 2 = disagree, 3 = somewhat disagree, 4 = neutral, 5 = somewhat agree, 6 = agree, 7 = strongly agree. The alpha reliability for this measure was 0.86.

#### Work Situation Awareness

The work situational awareness scale (Sneddon et al., 2013), consists of 20 items relating to situational awareness at work, e.g., “I find it difficult to concentrate for long periods of time” and “I find it difficult to keep track of everything that is going on around me.” Each item is scored on a 5-point rating scale; 1 = very often, 2 = quite often, 3 = occasionally, 4 = very rarely, 5 = never. The scale is reported to have good validity and reliability (Sneddon et al., 2013) and Cronbach’s alpha within the present sample was 0.88.

### Results and Discussion

Bivariate analysis revealed significant positive correlations between work-related rumination and cognitive failures (*r* = 0.33, *p* < 0.001) supporting H1, and indicating that as people ruminate about work, they also report making more cognitive failures. Also as expected, work-related rumination was negatively correlated with both cognitive flexibility (*r* = -0.24, *p* < 0.001), and situational awareness at work (*r* = -0.28, *p* < 0.001), supporting H2 and H3. Thus, the more people think and ruminate about work-related issues outside of work, the more they report less cognitive flexibility, and less awareness of what is going on around them at work. Together, these results support our proposition that work-related rumination is associated with executive functioning.

## Study 2: Work-Related Rumination and Executive Control

The aim of the second study was to replicate the findings of study 1 but to examine within a larger data set the association between levels of work-related rumination and specific types of cognitive failures in more detail. It was predicted that:

H1: Work-related rumination will be positively correlated with cognitive failures. Moreover it was predicted that medium and high work-related rumination would be associated with an increased likelihood of reporting cognitive failures.

### Sample and Participants

The sample was comprised of 939 working adults (Females = 53.3%) with an age range of 19–71 years (*M* = 42.91, *SD* = 9.66). The majority of participants (83.3%) worked full-time, and the average number of hours worked per week was 44.7 (*SD* = 9.64). Participants were represented from a range of occupations including: education, the emergency services, legal, nursing/health care, administration, management, medicine, and human resources. Participants were recruited via emails to organizations known to the research group. These public and private sector organizations spanned multiple industries including: pharmaceuticals, media, energy, banking, education, emergency services, and healthcare.

### Measures

#### Work Related Rumination and Cognitive Failures Were Assessed with the Same Measures Used in Study 1

There have been some inconsistencies with respects to the factor structure of the CFQ. Indeed, this was noted in Broadbent et al.’s (1982) original paper, where the authors suggest that the exact factor structure is highly dependent on the sample. This conclusion has been supported by others (e.g., Wallace et al., 2002). Therefore, a factor analysis was performed on the 25 cognitive failure items within the present sample. Three clear factors emerged with Eigen values greater than one, together accounting for 52.27% of the variance. A direct oblimin rotation was performed, variables were loaded on a single factor on the basis of the highest score. Items with a loading greater than 0.4 were retained. The first factor labeled ‘Lapses of attention’ contained six items (e.g., “Do you fail to notice signposts on the road”) had an Eigen value of 9.42 and accounted for 37.7% of the variance. The internal consistency (Cronbach α) of this factor was 0.78. The second factor labeled ‘Lack of focus of attention,’ consisted of five items (e.g., “Do you fail to listen to people’s names when you are meeting them?”) had an Eigen value of 1.4, and accounted for 5.68% of the variance (Cronbach α = 0.78) and the third factor, consisting of six items, labeled ‘Absent mindedness’ (e.g., “Do you find you forget why you went from one part of the house to the other”) accounted for 4.63% of the variance, and had an Eigen value of 1.15 (Cronbach α = 0.83). Four items were omitted as they had equal loadings across two or more factors. The factor structure broadly supported the findings of Wallace et al. (2002), although not exactly, as there were some differences.

### Results and Discussion

Bivariate analysis revealed a significant positive correlation between work-related rumination and total cognitive failures (range *r* = 0.34–0.41, *p* > 0.001) supporting the findings of the first study. To glean a greater understanding work-related rumination was divided into tertiles (low, medium, high), and each of the cognitive failures factors were divided into low and high groups. This method allows an examination of a ‘dose-response’ type association between levels of rumination and cognitive failures. Odds ratios and 95% confident intervals (CI) were calculated for each stratum of work-related rumination using low ruminators as the comparator group. A second set of models were calculated controlling for the effects of age, gender, job demands, and hours worked. As can be seen in **Table 1**, the likelihood of reporting cognitive failures was greater in the medium and high work-related rumination group, relative to the low group, with the greatest odds ratio in the high rumination group. This finding was consistent for the three factors of cognitive failures. Total cognitive failures revealed the greatest ORs in the high compared to the low rumination group (ORs, 5.09, CI, 4.19–8.32). There was little change in the ORs after adjusting for age, gender, job demands, and hours worked. In summary, these findings lend further support to the notion that work-related rumination is consistent with executive functioning issues.

**Table 1:** Odds ratios for work-related rumination and cognitive failures.

Cognitive failures factor and rumination group	(%)	*N*	Cognitive failures group		Crude OR (95% CI)	Adjusted OR^a^ (95% CI)
			Low	High		
*Lapses of attention*		
Low	34.4	323	241	82	1.00	1.00
Medium	29.6	278	142	136	2.81 (1.99–3.96)	2.83 (1.97–4.08)
High	36.0	338	128	210	4.82 (3.45–6.72)	4.81 (3.35–6.92)
*Lack of focus of attention*		
Low	34.4	323	247	76	1.00	1.00
Medium	29.6	278	162	116	2.37 (1.63–3.30)	2.33 (1.67–3.53)
High	36.0	338	164	174	3.44 (2.46–4.81)	3.63 (2.53–5.18)
*Absent mindedness*		
Low	34.4	323	245	78	1.00	1.00
Medium	29.6	278	165	113	2.15 (1.51–3.05)	1.93 (1.34–2.78)
High	36.0	338	142	196	4.36 (3.10–6.05)	3.96 (2.78–5.64)
*Total cognitive failures*		
Low	34.4	323	239	84	1.00	1.00
Medium	29.6	278	137	141	2.98 (2.08–4.12)	2.69 (1.87–3.88)
High	36.0	338	112	226	5.74 (4.10–8.03)	6.20 (4.28–8.90)

*OR, odds ratio; CI, confidence interval.*

*^a^Adjusted for age, gender, job demands, and hours worked.*

## Study 3: Work-Related Rumination and Executive Control

In the final study, we analyzed interview data from The Adult Psychiatric Morbidity Survey (APMS) 2007 a household survey conducted in England to examine the association between work-related rumination and executive control. Six proxy executive control items, namely: (1) trouble wrapping up the fine details of projects, (2) difficulty getting things done in order when tasks require organization, (3) problems remembering appointments or things, (4) avoid or delay getting started, (5) fidget or squirm when have to sit for long time, and (6) feel overly active and compelled to do things, were examined.

H1: Levels of work-related rumination will be positively associated with self-reported increased deficits in executive functioning as assessed by the six proxy items.

### Methods

Between October 2006 and December 2007, the National Centre for Social Research (NatCen) conducted The Adult Psychiatric Morbidity Survey [APMS] (2007) to assess the prevalence of psychiatric morbidity in private households within England. In total 7461 interviews were conducted. Each questionnaire item is presented and replied to verbally, by each participant who are individually interviewed. For a full description of the survey McManus et al. (2009). Participants in the present study were a subset of 2460 adult workers from a range of occupations, who reported they were in full-time employment. Their age ranged from 16 to 70 years (mean 42, *SD*, 11.9), and 59.7% of the sample were male.

#### Measures

##### Executive functioning

Executive functioning at work was assessed using the six items reported above. Originally, the six items have been used to assess levels of ADHD, however, these items have been adapted to be used within the occupational environment to assess cognitive performance at work (Kessler et al., 2005, 2009). Items were rated as: 1 = never, 2 = rarely, 3 = sometimes, 4 = often, 5 = very often.

##### Work related rumination

Following Cropley and Zijlstra (2011), work related rumination was computed from the over commitment items of the Effort-Reward Imbalance Questionnaire (Siegrist et al., 2004), using the following items: “As soon as I get up in the morning I start thinking about work problems;” “When I get home, I can easily relax and switch off work” (reversed), and “Work rarely lets me go, it is still on my mind when I go to bed”). Items were rated on a 4-point scale, 4 = strongly agree, 3 = slightly agree, 2 = slightly disagree, 4 = strongly disagree. A mean score was calculated where higher values indicated greater rumination. The internal consistency (Cronbach α) of the unwinding factor was 0.80.

### Data Analysis

As in study 2, to demonstrate the association between different levels of rumination and executive control, work-related rumination was divided into tertiles (low, medium, high) and the six analog executive control items were divided at their respective median. Crude odds ratios were initially calculated and then age, gender, anxiety, and depression were included as covariates.

### Results and Discussion

**Table 2** summarizes the odds ratios for the six executive control items and work-related rumination. As can be seen, relative to low rumination, high rumination was associated with increased ORs for all six analog executive control items. These ORs ranged from 1.84 to 2.42, indicating that high ruminators were approximately twice as likely to experience poor issues of executive control. These findings were not overtly changed once the covariates were added. In the medium rumination group, four of the six proxy executive control items showed an increase ORs compared to the low rumination group, however, ‘difficulty getting things done in order when task require organization,’ and ‘feel overly active and compelled to do things,’ were no different to the low rumination group. Once the covariates were taken into account, ‘fidget or squirm when have to sit for long time,’ was reduced to non-significance.

**Table 2:** Odds ratios for work-related rumination and ADHD items.

ADHD items and rumination group	(%)	*N*	ADHD group		Crude OR (95% CI)	Adjusted OR^a^ (95% CI)
			Low	High		
*Trouble wrapping up the fine details of projects*		
Low	27.8	682	542	140	1.00	1.00
Medium	38.0	935	684	251	1.42 (1.12–1.79)	1.39 (1.10–1.76)
High	34.2	841	517	342	2.42 (1.92–3.06)	2.21 (1.73–2.81)
*Difficulty getting things done in order when task requires organization*		
Low	27.8	683	584	99	1.00	1.00
Medium	38.0	935	781	154	1.16 (0.88–1.53)	1.10 (0.83–1.46)
High	34.2	841	641	200	1.84 (1.41–2.40)	1.50 (1.13–1.98)
*Problems remembering appointments or things*		
Low	27.8	683	543	140	1.00	1.00
Medium	38.0	936	683	253	1.43 (1.13–1.81)	1.35 (1.06–1.75)
High	34.2	841	529	312	2.28 (1.81–2.88)	2.01 (1.58–2.52)
*Avoid or delay getting started*		
Low	27.7	683	483	200	1.00	1.00
Medium	38.1	936	581	355	1.47 (1.19–1.82)	1.43 (1.16–1.78)
High	34.2	840	427	413	2.33 (1.88–2.89)	2.06 (1.65–2.57)
*Fidget or squirm when have to sit for long time*		
Low	27.8	683	397	284	1.00	1.00
Medium	38.1	935	490	455	1.27 (1.04–1.54)	1.21 (0.98–1.48)
High	34.1	836	351	485	1.93 (1.57–2.37)	1.76 (1.42–2.18)
*Feel overly active and compelled to do things*		
Low	27.8	683	469	214	1.00	1.00
Medium	38.0	934	604	330	1.19 (0.97–1.47)	1.16 (0.94–1.43)
High	34.2	840	437	403	2.02 (1.63–2.49)	1.87 (1.50–2.34)

*OR, odds ratio; CI, confidence interval.*

*^a^Adjusted for age, gender, anxiety, and depression.*

## General Discussion

Attributes relating to executive functioning — attention, cognitive flexibility, and planning — are crucial qualities if workers wish to succeed in the workplace. Deficits in executive functioning can make it particularly difficult for workers to perform and complete tasks that require mental control. Across three independent studies this paper consistently demonstrated that work-related rumination is associated with deficits in executive functioning. Work-related rumination was positively and significantly associated with self-reported cognitive failures, and reduced situational awareness at work, and negatively associated with cognitive flexibility. In addition, work-related rumination was positively associated with trouble wrapping up the fine details of projects, difficulty getting things done in order when task require organization, problems remembering appointments or things, avoid or delay getting started, fidget or squirm when have to sit for long time, and the feeling of being overly active and compelled to do things. Thus, high ruminators show deficits in the key skills required for productivity and goal directed behavior in the workplace.

The majority of previous studies that have examined work-related rumination/psychological detachment from work, have done so in relation to health issues (Sonnentag and Fritz, 2015). To our knowledge, this is the first study that has investigated the effects of work-related rumination and executive functioning within an occupational context. While different methodologies – survey and interviews – were utilized in the present study, the findings are nonetheless based on self-report. Although ipso facto, rumination can only be assessed by self-report, it would be of interest to replicate the results using objective measures of executive functioning such as computer based cognitive programs or card sorting tasks (Monchi et al., 2001; Strauss et al., 2006). The self-report nature of the measures also leads to the possibility of reporting biases associated with common method variance. Within the context of these limitations, the results nonetheless, suggest that thinking about work outside of work, increases the risk of making errors or mistakes at work.

Our proposition is that rumination affects executive functioning, leading to a reduced cognitive capacity, and the findings are consistent with this. Ruminating about work could deplete executive resources leading workers to be less focussed and flexible in their thinking and cognition. That is, rumination consumes resources that would otherwise be directed to the task at hand. It has been speculated that the act of ruminating appears to temporarily take the prefrontal cortex ‘oﬄine’ thereby interfering with executive functioning (Ottaviani et al., 2009). Our findings, however, are also compatible with the notion that depleted executive resources increases the likelihood of ruminating. People who display less executive control could be more prone to making errors and mistakes at work, and therefore more likely to ruminate about them when not at work. Similarly, if people have depleted executive control, their mind is more likely to wonder and they will have difficulty concentrating and focussing on tasks. This finding is supported by the clinical literature (Watkins and Brown, 2002; Whitmer and Banich, 2007; Mor and Daches, 2015). We were not actually able to address the issue of causality in the reported studies. Although our findings are fully compatible with the notion that ruminating about work reduces executive functioning, or vice versa, we do not actually demonstrate this causally. The association between rumination and executive functioning could be caused by a third unknown variable, or indeed by workload/work pressure.

Future research needs to address whether executive functioning deficits precedes the onset of rumination or occurs as a result of rumination. It would also be of interest to examine whether executive functioning issues increases the likelihood of, or makes people vulnerable to rumination about work. Vulnerability may change, when work is particularly demanding, or when people become fatigued. Whether executive function issues persist after the remission of rumination is another important question and could be tested over-time in longitudinal study designs.

The present findings nonetheless may have implications for the design of intervention programes. Occupational health interventions are typically directed toward reducing work demands or aim to provide individuals with coping resources to help them deal with work demands. Creating a healthy working environment should lead to a reduction in depleted executive resources. In respect to work-related rumination for example, workers may be provided with techniques or strategies to help them unwind post work (Cropley, 2015). Interventions in the form of CBT training or mindfulness (Hülsheger et al., 2014; Querstret et al., 2015, 2016) have been shown to reduce the frequency and intensity of work related thoughts. The present findings suggest that future interventions aiming to reduce work-related rumination should also address the issues relating to central executive functioning. Although the evidence surrounding the efficacy of different methods for strengthening executive functioning is equivocal, there is strong evidence of the benefits of exercise in this respect (Guiney and Machado, 2013). Exercise interventions either work or home based could be a cost effective way of improving overall health and wellbeing but also for increased executive control. However, this is mere speculation, and whether reductions in work-related rumination is actually mediated by improved executive functioning needs to be empirical tested in future research.

## Author Contributions

MC and FZ developed the original idea for this paper. DQ provided data for study 2, and SB developed the idea and conducted the background work for study one. MC and FZ drafted the paper with additional input and revisions/interpretation from DQ and SB. All authors have agreed the final version of the submitted manuscript and have all made an intellectual contribution.

## Conflict of Interest Statement

The authors declare that the research was conducted in the absence of any commercial or financial relationships that could be construed as a potential conflict of interest.
